# Digital Articulation: Examining Text-Based Linguistic Performances in Mobile Communication Through Keystroke-Logging Analysis

**DOI:** 10.3389/frai.2020.539920

**Published:** 2021-01-25

**Authors:** Joel Schneier

**Affiliations:** Department of Writing and Rhetoric, University of Central Florida, Orlando, FL, United States

**Keywords:** keystroke analysis, mobile communication, paralinguistic cues, digital articulation, text messaging, computational sociolinguistics

## Abstract

This study examines how text-based mobile communication practices are performatively constructed as individuals compose messages key-by-key on virtual keyboards, and how these *synchronous performances* (Mobile interface theory: embodied space and locative media. New York, NY: Routledge) reflect the iterative process of constructing and maintaining interpersonal relationships. In doing so, this study reports on keystroke-logging analysis (see Writ. Commun. 30, 358–392) in order to observe how participants (*N =* 10) composed text as part of everyday mobile communication for the period of one week, subsequently producing 179,996 individual keystroke log-file records. Participants used LogKey, a virtual keyboard application made exclusively for this study to run on the Android mobile operating system. Analysis of keystroke log-file data suggest that timing processes of composing text-messages may differ as participants messaged with different categories of interlocutors, composed on different communication applications, and composed paralinguistic features—such as variants of *Lol* and *Haha* Thurlow and Brown, (Discourse Anal. Online, 2003, 1, 1); Tagg, (Discourse of text messaging. 2012, Bloomsbury, UK)—at different turn-taking positions. This evidence suggests that keystroke-logging methods may contribute to understanding of how individuals manage interpersonal relationships in real-time (Please reply! the replying norm in adolescent SMS communication,” in The inside text: social, cultural and design perspectives on SMS. (Norwell, MA: Springer), 53–73); (Beyond genre: closings and relational work in texting,” in Digital discourse: language in the new media. (Oxford: Oxford University Press), 67–85), and suggests future direction for methodologically studying linguistic performances as part of text-based mobile communication.

## Introduction

The increasing ubiquity of mobile technology in recent decades has given rise to forms of sociolinguistic research that have explored how text-based language may be may be used as part of everyday interpersonal discourses in text-messaging (see Ling and Yttri, 2001; Thurlow and Brown, 2003; Spilioti, 2011; Tagg, 2012), as well as perform social identities in social media (see Pavalanathan and Eisenstein, 2015a; Jones, 2015). Computational methods, such as Grieve, Nini, and Guo (2017) and Pavalanathan and Eisenstein (2015b), have similarly demonstrated the value in examining how text-based linguistic features may be transmitted and diffused across online social networks to be made part of individual and social performances (Coupland, 2007; Androutsopoulos, 2014b) via broadcast mechanisms available in everyday mobile telephony, particularly social networking sites such as Twitter. In this way, tracing how language may be traded in through online interactions, conducted at the touch of a screen, has provided sociolinguists a front-row seat to witness language diffusion as it occurs in real-time across the Twitter-verse (see Jones, 2015; Grieve et al., 2017).

While these methods have yielded insights into how linguistic forms may be diffused across online social networks, as well as suggest how individuals may adopt and use newly enregistered features for performing social identities, lost among these methods is the notion of a flesh-and-blood performer of language. After all, the underlying assumption in quantitatively and computationally examining the ‘firehose’ of linguistic data on Twitter is that individual humans were responsible for composing said tweets, and that such performances reflect real-life social and linguistic meaning (Brock, 2020; Eisenstein, 2013; Jones, 2015). This study therefore asks, *how can we examine how individuals use mobile technology to compose text-based linguistic features in real-time?* And,* how do the timing processes of composing these linguistic features through mobile media demonstrate how individuals perform social identities?* I therefore present a methodology that examines how text-based mobile communication practices are composed in real-time through keystroke analysis, and suggest that such a methodology, alongside established computational methods to examine the large scores of public text-based data, contributes to a stronger understanding of how mobily-mediated linguistic performances meaningfully unfold in individuals’ everyday lives.

This study therefore reports on keystroke-logging data (see Leijten and Van Waes, 2013) as part of observations of how participants (*N* = 10) composed text as part of everyday mobile communication for the period of one week, subsequently producing 179,996 individual keystroke log-file records. Participants used LogKey, a virtual keyboard application developed exclusively for this study to run on the Android mobile operating system. This small study therefore served as a preliminary test of the feasibility of using LogKey to conduct keystroke analysis on individuals’ own mobile devices, as well examine this study’s stated questions. Analysis of keystroke log-file data obtained from this study yielded insights suggesting that timing processes of composing text may differ as participants messaged with different interlocutors, compose text for different mobile applications, and composed paralinguistic cues—such as variants of *Lol* and *Haha* (Thurlow and Brown, 2003; Tagg, 2012)—at different discursive positions in a text-message. I argue that the findings from this small study, while not generalizable, may contribute to stronger understandings of how individuals manage interpersonal relationships in real-time through composing, sending, and receiving text through mobile communication (Ling and Yttri, 2001; Thurlow and Brown, 2003; Laursen, 2005; Spilioti, 2011). Further, this study’s use of LogKey to examine individuals’ everyday text-based linguistic performances in mobile communication over the period of one week represents one of the first—if not the first—to do so, and therefore suggests future direction for methodologically studying how individuals meaningfully produce and disseminate written language through mobile devices in real-time.

The following section will offer a theoretical framing for how text-based language may take on and perform social meaning, as well as review recent research that has to examined text through sociolinguistic, computational frameworks, and keystroke analysis. This background section will be followed by an overview of this study’s methods, followed by an overview of keystroke data collected, analysis of keystroke data in combination with discursive contexts in which those keystrokes occurred, and will conclude with a discussion that will suggest further study and directions for developing methods to examine text-based linguistic practices in mobile media.

## Theorizing and Tracing Text-Based Language

This section will briefly provide a theoretical framework for studying text-based language in order to identify the exigence of this study's methodological contributions. In doing so, I do not intend to provide an exhaustive review of literature in this area; rather, I intend to present a rationale for incorporating keystroke-logging methods to ask sociolinguistic questions about text-based performances through mobile media. I argue that it is useful to examine text-based language in communicative media through two underlying assumptions. The first assumption is that the composition and transmission of such text is part of individual social *performances* in individuals’ lived experiences. This performative conceptualization, which notably draws upon Goffman (1956), Butler (1997), and Coupland (2001), frames language use as continually constructing social identities, and each performance may contribute to meaning-making. Under this assumption, composing and sending text-based language through mobile media reflects how individuals perceive their own social identities and how they want to be perceived by their interlocutors.

The second assumption is that linguistic units transcribed into text can serve as material forms of *symbolic capital* that can signal social meaning and value when used as part of everyday practices (Bourdieu, 1984). For example, Eckert (2001) has argued that as adolescents navigate competing ideas of peer groups, adults, and their wider communities, they are “mutually engaged in the production of new meaning” (p. 34–35) through use of linguistic resources that form symbolic capital to *style* themselves according to one type of identity or another. Eckert (2008) further argued that through the very trading in their use, symbolic capital—linguistic or otherwise—may become associated with a variety of different possible social meanings, which Eckert terms the *indexical field*. Androutsopoulos (2014b) additionally argues that once a linguistic performance is recorded in audio-, visual-, or text-based media, that it is available material that individuals may use as stand-ins for symbolic capital, what he calls *media fragments*. These *media fragments* may become highly symbolic to trade in as part of performing social identities for particular peer groups (Georgakopoulou, 2014). Indeed, just as Eckert (2001) argued that clothing may signal one social identity or another among adolescent peer-groups, exchanging media fragments through frequent text-messages (Ling and Yttri, 2001; Laursen, 2005), emoticons or emojis (Baron and Ling, 2011; Highfield and Leaver, 2016), Facebook comments (Androutsopoulos, 2014a; Androutsopoulos, 2014c), or even YouTube videos (Georgakopoulou, 2014) may all serve to signal social identities, relationships, and even meanings that are continually being performed and negotiated.

These two assumptions frame text-based language-use as symbolic capital for performing social meaning, which may therefore more contribute to language change, for example, as media fragments are sent and received through various forms of media. Coupland (2001) has argued that in purposefully performing one style or another through broadcast media, such as radio, may in fact *produce* a shift in that meaning as they play with and balance perceptions of their audience and themselves. When these performances are received by audiences, it may change the indexical field of meanings attached to linguistic features because said linguistic features as well as their social meanings are diffused through broadcast media simultaneously. Coupland (2007) calls this process of new social and linguistic meaning developing through diffused broadcast media *decontextualization*, and Androutsopoulos (2014b) argues that decontextualized language may take on a range of new potential meanings that were construed through the broadcast and the audience’s preconceived notions, a process he calls *recontextualization*. According to Androutsopoulos (2014b) recontextualized language reconfigures the ideological linkages in the indexical field of meaning.

This process of de- and re-contextualization may be readily observable through text-dominant mobile media, for example the rapid diffusion of *(on) fleek* on Vine and then Twitter (Grieve et al., 2017), and may be evidenced, as with spoken language, at both the social and individual level. In other words, the occurrence and prevalence of linguistic features might reflect broader social meaning, but the manner in which it is composed in real-time by individuals may reflect how, to paraphrase Coupland (2001), the individual is performed through the social. As I suggest later, understanding the processes through which individuals compose linguistic units into text may therefore be indicative of how they function to perform social meaning. The following section will therefore discuss how the production of text-based linguistic units may be seen as being performed in real-time, how paralinguistic cues, such as *Haha* and *Lol,* may evidence these performances, and how keystroke logging methods may aid in better understanding these performances.

### Text and Time

Since the start of the new millennium various researchers at the intersection of sociolinguistics and media studies have documented the various social functions of text-based language, and how spoken communication practices are adopted for text-based media (see Ling, 2008). For example, researchers have observed how individuals use frequent text messaging to maintain contact (Ling and Yttri, 2001; Laursen, 2005), reconstitute paralinguistic meaning (Thurlow and Brown, 2003), adopt politeness strategies (Spilioti, 2011), circulate multimedia (Georgakopoulou, 2014), establish discursive structures and meanings for emojis (Sampietro, 2016; Pérez-Sabater, 2019), and even creatively play with spelling (Tagg et al., 2012; Tagg, 2012). Scholars examining social media have additionally examined how individuals may strategically modulate their audiences (Androutsopoulos, 2014a; Pavalanathan and Eisenstein, 2015a), perform social identities (Pavalanathan and Eisenstein, 2015b; Jones, 2015; Brock, 2020), pair with audio-visual channels (Piwek and Joinson, 2015; Highfield and Leaver, 2016and), or even participate in social media trends (Grieve et al., 2017). It is therefore noteworthy that scholars in the last 2 decades have identified—and continue to identify—the numerous ways that text-based language may be linked to different social meanings and functions that are continually negotiated through the technological affordances of the media through which text is circulated. An important example of this is the practice of what Thurlow and Brown (2003) term *paralinguistic restitution*, wherein individuals actively use the material affordances of the medium in order to communicate paralinguistic information, such as emotion or tone, that may be otherwise transmitted via prosodic features in spoken communication. In text-messaging, examples of paralinguistic restitution can include capitalization or reduplication to indicate stress (Thurlow and Brown, 2003; Fuchs et al., 2019), emoticons or emojis to indicate gesture (McCulloch, 2019), and *Haha* or *Lol* to indicate *shared laughter* (Jefferson, 1979; Ling and Yttri, 2001; Baron and Ling, 2011).

Further, corpus-based and computational methods, particularly for examining data from Twitter or text-messaging, have provided the opportunity to quantitatively aggregate and analyze text-based data in relation to broader sociolinguistic variables. As demonstrated by Eisenstein et al. (2014), Nguyen et al. (2015), Jones (2015), and Pavalanathan and Eisenstein (2015a), among others, how Tweeters tweet may reflect the configuration of their online social networks as well as regional demographic configurations of the geographic area from where they tweet (Jurgens, 2013; Eisenstein et al., 2014; Pavalanathan and Eisenstein, 2015b). In other words, what Tweeters tweet may reflect traditional “real-world” networks, communities, and online social identities.

While it is beyond the scope of this paper to fully survey and summarize the breadth of recent research in the emerging discipline called “computational sociolinguistics” (Nguyen et al., 2016), I do wish to echo and examine questions raised by Nguyen et al. (2016) that drive at the heart of linguistic research: *how do we locate individual agency in such data?* In their exhaustive survey of emerging computational sociolinguistic research over the last decade, Nguyen et al. (2016) argues that a central challenge of the nascent discipline is to reconcile the macro-scale informational and structural dimensions yielded from corpus research of text-based linguistic data with the real-world social performances and decisions of a flesh-and-blood person. Indeed, Nguyen et al. (2016) suggest that, in addition to examining traditional sociolinguistic variables, researchers can strive to further locate individual agency by incorporating “multimodal data” (p. 575). I suggest that such possible data modes and channels could detail how text is inscribed into a medium in real-time, as such data would illuminate the social, cognitive, and physiological processes through which text-based language is *articulated*. This form of *digital articulation* could include temporal and log-file about how individuals use their hands to input text to produce written language. Such data could complement computational methods to examine the widespread use of prevalent linguistic feature in Twitter and texting in order to better understand how those features are performed and meaningful to individuals.

Some researchers at the intersection of this field have indeed posited the connection between text and speech-based articulatory processes. Eisenstein (2013) and Eisenstein (2015) has examined numerous grapho-phonological “respellings” such as t/d-deletion or g-dropping for ing morphemes. Eisenstein (2015) argues that “when alternative spelling is linked to phonetic variation, it acquires at least the residue of the systems of phonological, grammatical, and social patterning present in speech” (p. 181); however, this may be dependent upon interactional contexts for addressing different audiences (see Pavalanathan and Eisenstein, 2015a), as syntactic and phonological constraints do not reliably predict variation in/ing and t/d-deletion in text as in speech. While Jones (2015) suggests that grapho-phonological variation on Twitter may reflect phonological realities of individual’s speech patterns, I argue, as with Eisenstein (2015), that grapho-phonological variation in text-based linguistic performances may only reflect a “residue” of an individual’s lived speech patterns rather than a verbatim transcription. The complex process of producing written language is drastically different from spoken language, as the interface of cognitive and articulatory processes involves overlapping *but materially different* physiological, psychomotor, interactional, and cognitive processes. Writing takes more time, requires psychomotor processes to control technological transcription, and results in asynchronous symbolic material. How individuals may compose, evaluate, or even strategically stylize text to satisfy their communicative needs may therefore be indicative of metalinguistic processes, and examining these processes may require theoretical and methodological consideration of how texts are produced. This study therefore follows Eisenstein’s (2015) suggestion to use insights from keystroke-logging methods in order to examine how language is *digitally* articulated in real-time, and will do so through an investigation of two paralinguistic features: *Haha* and *Lol*. These features will be discussed in further detail in the next section.

### Paralinguistic Features in Mobile Communication: Or, Who Laughs for Thee?

As noted above, individuals communicating through text-based media, such as text-messaging, may creatively use the affordances of the medium to engage in *paralinguistic restitution* in order to communicate information about paralinguistic features such as tone, stress, etc. (Thurlow and Brown, 2003). Spilioti (2011) offers a vivid example of *paralinguistic restitution* through analysis of closings in text-messages (e.g., *bye!* or *xoxo*), observing that, while closings may be typically absent from text-messages, the presence of closings may serve to strategically mitigate perceived face-threats via Brown and Levinson’s (1987) politeness framework and signal relational closeness. The use of closings to communicate paralinguistic information is therefore predicated upon its expected norms (i.e., absence) related to everyday texting among interlocutors, which suggests that use of textual linguistic features may reconstitute paralinguistic meaning based upon frequency of use as well as how they are used within a text message. This study will examine two such paralinguistic features, *Haha* and *Lol*, and the remainder of this section will provide a brief review of these features and justify their selection for analysis in this study.

*Haha* and *Lol* may be seen as text-based representations of reacting with humor: *Haha* is a grapho-phonological approximation of laughter that has been recorded as far back as 1000 in Ælfric’s *Grammatik und Glossar* (Ælfricof and Zupitza, 1880); while *Lol* is an acronym standing for “laugh out loud” that was possibly first coined in English-speaking internet chatrooms from the 1980s (McCulloch, 2019). Both features have been well-documented in digitally-mediated communication (DMC) research, for example, in computer-based writing registers such as Instant Messaging (Baron, 2004; Lewis and Fabos, 2005; Tagliamonte and Denis, 2008; Haas et al., 2011), and may be textually realized in a number of ways that conform to other common processes, such as reduplication or capitalization for emphasis (e.g., *hahahaha* or *LOLOL*). Regardless of whether *Haha* and *Lol* may be considered different variants of the same variable or different variables entirely (see Tagliamonte and Denis, 2008; Tagliamonte, 2016), both features appear to have maintained relatively stable usage across multiple studies over the last 2 decades. For example, Baron (2004) observed *Lol* made up 0.6% of all words in a corpus of IMs, Tagliomonte and Denis’s (2008) found that variants of *Lol* and *Haha* made up, respectively, 0.41% and 1.47% of all words in a corpus of IMs, and Tagliamonte, 2016 similarly found that variants *Lol* and *Haha* made up, respectively, 0.69% and 0.40% of all word units in a corpus of IMs, text-messages, and other e-messages. As suggested by Tagliomonte (2015), these features have become present across other DMC registers, particularly *mobile* registers such as texting (Thurlow and Brown, 2003; Laursen, 2005; Baron and Ling, 2011) and even social media like Twitter (Pavalanathan and Eisenstein, 2015a), which is commonly interfaced through mobile devices (Brock, 2020).

The frequency and widespread usage across registers therefore suggests that *Haha* and *Lol* are relatively established and stable paralinguistic features in various registers of DMC (at least in the Western, English-centric world). Indeed, as will be further detailed below, participants in this study used these features with similar relative frequencies as noted above. I therefore argue that, because of the frequency with which these paralinguistic features are used, they are ideal to examine the usefulness of using keystroke-logging analysis to ask sociolinguistic questions. After all, the more frequently particular features are used by individuals on an everyday basis, the more behavioral keystroke data can be analyzed to examine articulatory patterns (e.g., how fast a feature is composed).

In addition to *Haha* and *Lol* being established paralinguistic features in texting, I suggest that they are also useful for examining interactional and situational contexts of texting. Thurlow and Brown’s (2003) study demonstrated that texting conversations tended to manage relational intimacy as well as coordinate social discourse and activity. Paralinguistic restitution, which includes use of paralinguistic features such as *Haha* and *Lol,* therefore serves as part of these broader interpersonal functions, and this may be further seen through the ways in which texters frequently use such paralinguistic features to continually validate their relationship to one another (Ling and Yttri, 2001; Laursen, 2005). Through this lens, *Haha* and *Lol* do not just represent literal laughter, but may, as Baron (2004) suggested, structurally serve as “phatic fillers for the equivalent of OK, cool, or yeah” (p. 411). Further, studies of various paralinguistic features in texting (Ling and Yttri, 2001; Highfield and Leaver, 2016; Sampietro, 2016; Pérez-Sabater;2019) have commonly observed that such features occur at turn-taking boundaries (i.e., the start or end of a text message), and, to a lesser extent, clause boundaries within a message. This suggests that *Haha* and *Lol* in texting might serve multiple intersecting functions: as symbolic capital to manage interpersonal relationships, and to coordinate turn-taking structures similar to *shared laughter* (Jefferson, 1979). From this vantage point, *Haha* and *Lol* indeed maintain some of the “residue” of spoken language as Eisenstein (2015) suggests, but also function according to the ways in which individuals negotiate the technological affordances and discursive expectations of text-messaging registers.

I further suggest, following Spilioti (2011), that Brown and Levinson’s (1987) politeness framework is powerful for sociolinguistic interpretations of the specific interactional contexts in which *Haha* and *Lol* may be used in texting. Within Brown and Levinson’s (1987) framework, individuals may use various politeness strategies during communication in order to mitigate possible face-threatening acts (FTAs) to themselves and interlocutors. According to Brown and Levinson (1987), FTAs can occur and be mitigated through linguistic, non-linguistic, or paralinguistic channels, and can affect an individual’s *positive face*, i.e., “the desire […] to be approved of” (p. 13), or *negative face*, i.e., “the desire to be unimpeded in one’s actions” (p. 13). For example, an individual may use *shared laughter* via a “laugh particle” (Jefferson, 1979) at the start of a turn in response to an interlocutor’s joke in order to avoid damage to the interlocutor's positive face (i.e., in order to preserve the interlocutor’s self-value of being humorous and liked); while laughter at the end of a turn may preserve the positive face of the speaker and mitigate negative face-threats to the interlocutor (i.e., in order to preserve the speaker's self-value and avoid imposing upon the interlocutor). As Spilioti (2011) suggested, since texting conversations and the asynchronous turn-taking structure serve to manage relational work, Brown and Levinson's theories frame every sent and received text message as symbolically imbued with politeness strategies. For example, since individuals are compelled to send messages frequently (Ling and Yttri, 2001) and respond to messages quickly (Laursen, 2005) to signal relational closeness, texting conversations may be seen as continually navigating politeness strategies because every sent message is a negative FTA (i.e., because it imposes on the receiver) and every response is a positive FTA (i.e, because it signals how the receiver is valued). Turn-taking positions in a text message (i.e., the start or end of a message) may therefore be seen as highly salient positions through which texters work to mitigate such FTAs, and the use of *Haha* or *Lol* at these turn-taking position may symbolically negotiate these politeness strategies.

Further, as noted by Meredith and Stokoe (2014), asynchronous text-based channels such as texting and IM afford individuals the ability to manage and even repair execution of these politeness strategies in the message space unseen by the interlocutor, i.e., prior to sending the message. Meredith and Stokoe (2014) found that such *message construction* repairs bear similarity to repair work in spoken language, albeit while remaining unseen and therefore “unaccountable for some interactional matters” (p. 202). This suggests that the seemingly unseen processes through which individuals cognitively select and compose specific textual features reflects how individuals strategically manage politeness strategies. The timing processes for composing *Haha* or *Lol* at different turn-taking positions, which are seen only by the individuals composing the message (Meredith and Stokoe, 2014), may reflect the *residue* of how individuals are cognitively processing these strategies in order to manage relationships with their interlocutors. This requires attention not only to how and where *haha* and *lol* are distributed in sent and received messages—which may be accomplished through text-based linguistic analysis—as well as the timing processes through which these features are composed—which may be accomplished through keystroke-logging analysis.

### Keystroke-Logging Analysis and Digital Articulation

In this section I will provide a brief contextualization of keystroke-logging analysis and how it may offer articulatory evidence for the linguistic production of text. Keystroke-logging analysis has roots in writing studies, an area of research that emerged in the 1980s and has pulled in researchers from various fields, such as cognitive science, applied linguistics, educational psychology, technical communication, etc (see Hayes, 1996; Cislaru, 2015). Central to this methodology is examining temporal data from writing in order to infer cognitive processes that are engaged *during* writing (Plane, 2015). This requires examining a textual artifact according to how it was composed in real-time, i.e. *synchronously*, in conjunction with the completed text as the primary source for extricating linguistic meaning. I use the term *digital articulation* purposefully, as the metaphor of articulation—which linguists often think of in physiological, perceptual, and acoustic terms—in order to call attention to the fact that composing linguistic material in text involves an articulatory process that unfolds synchronously (Farman, 2012; Plane, 2015). This bears some similarity to speech, except that writing involves digital^1^ articulatory mechanisms and results in an asynchronous textual artifact. This differentiation from speech therefore requires writing researchers to unpack what Grésillon and Perrin (2015) term the *double black box*, i.e., the processes through which a text was composed as well as how those processes evidence cognitive and social processes involved in composition.

Keystroke logging therefore serves as one such methodology to unpack this *double black box*, as it allows analysis of temporal records of discrete input-based events involved in writing with a computer, i.e., pressing individual keys on a keyboard in order to compose a digital text. Leijten and Van Waes (2013) argue that keystroke logging allows researchers to both re-construct the temporal processes through which individuals composed a text *and* to observe writers rather unobtrusively. Further, because keystroke logging’s primary unit of analysis is discrete keystroke events, temporal analysis is located primarily in *pauses*, i.e., the time in *between* the input of individual keystrokes. This conceptualization of the *pause* borrows heavily from speech production, in which, as argued by Miller (2006), pausing during writing provides indirect and inferential evidence of writers’ cognitive resources, including attention management and long-term memory retrieval.

Pause-based data has therefore been shown to relate to linguistic characteristics (Van Waes, Leijten, Lindgren, and Wengelin, 2015). This requires coordinating pause-based analysis, typically through the measurement of time between key presses called the *inter-key interval* (Leijten and Van Waes, 2013), as well as the processual sequence of keystrokes that construct recognizable linguistic units of information. For example, a given sequence of keystrokes, such as [H], [e], [l], [l], [o], therefore may represent the intended construction of the word *Hello*, and the inter-key intervals for the first key may therefore be longer than the intervals for all subsequent keys. Using keystroke analysis to examine linguistic content is therefore, in some ways, similar to using acoustic analysis in order to examine phonological variables such as speech rate (see Kendall, 2013), as pause-times will often distinguish between smaller and larger chunks of linguistic units. For example, the pause between [e] and [l] in *Hello* will be shorter than between [o] and the first letter of the next word (Van Waes et al., 2015).

Researchers have therefore taken multiple approaches in order to examine linguistic segments, particularly latencies between different syntactic units, morpho-phonological syllable boundaries (see Nottbusch, 2010), as well as use specific keystroke events and pause times in order to distinguish boundaries between *pause bursts* (i.e., keystroke activity between pauses over 2000 milliseconds) or *revision bursts* (i.e., keystroke activity relating to revising text) (see Galbraith and Baaijen, 2019). For example, using *Inputlog* ^2^, Leijten et al. (2012) incorporated various NLP tools on linear notation of keystroke data (called S-notation) that represents the non-linear process of composing textual products. Leijten et al. (2012) argues that this allows analysis of word-level revisions (i.e., individual words that are revised for individual characters), deleted segments (i.e., multi-word units that are deleted within the same sequence of deletion activity), and the final text; all of which are subject to part-of-speech (PoS) tagging, lemmatization, chunking, and word frequency analysis. In addition, Olive and Cislaru (2015) combined both *Inputlog*’*s* NLP analysis to compare with corpus-based methods to examine the timing processes of *pause bursts* as well as *repeated segments* (i.e., a sequence of two or more linguistic units that occur at least twice in a corpus), and found evidence that only 3% of these units shared overlapping syntactic structures. Further, in examining text produced by college students taking a test (*N =* 38), Plank (2016) additionally found that, when applying a Long-Short Term Memory model (a type of bi-directional neural network), pauses helped illuminate chunking at the word-level, but not necessarily the morpho-syntactic level. This research demonstrates that keystroke-log data may be organized in a number of ways in order to analyze recognizable linguistic units of information, particularly temporal analysis surrounding word boundaries.

Further, considering the idiosyncratic nature of individual writers (Plank, 2016) and variations across written registers individuals may be familiar with/have access to (Biber and Conrad, 2009), keystroke analysis has demonstrated the value in looking more qualitatively at individual writers in order to more robustly examine how text is produced in context. For example, in examining writers with dementia, Leijten et al. (2015) found that such writers required much more time than non-dementia writers of similar ages to compose nouns and verbs compared to articles or prepositions, which the authors suggest reflects the greater cognitive demands placed on writers with dementia. Leijten et al. (2019) applied similar methods to compare native Dutch speakers writing in Dutch (L1) and English (L2), observing that pause-based differences may be attributed to language, word length, and PoS, and that these pause patterns may repeat for frequent two- and three-word constituents. Importantly, Leijten et al. (2019) also observed that, based on pause-based analysis, language differences primarily were limited to spelling and word choices. Leijten et al. (2014) additionally demonstrated that examining even a single writer producing a single text over a several-day period may yield important theoretical insights about how writers may use schematic knowledges of various genres and registers to construct texts. Most recently, Bowen and Van Waes (2020) used keystroke logging and systemic functional linguistics frameworks in order to examine revisions during writing, including the finding that revisions may most frequently occur at or just before the point of inscription. While Bowen and Van Waes (2020) also only observed a single writer composing over multiple writing sessions, their study demonstrated the rich possibilities of applying keystroke analysis in order to examine linguistic frameworks. Indeed, the amount of data obtained via keystroke logging from individual writers over longer spans of time, rather unobtrusively and indirectly, allows for in-depth analysis and consideration of how individuals may meaningfully construct written language in context in order to contribute to theory-building to conduct broader and more generalizable studies.

While only a short sample, these examples demonstrate both the value of using keystroke analysis to ask linguistic questions regarding text production. These studies may privilege lexical and morpho-syntactic analysis, partially due to the incorporations of NLP methods; however, as suggested by Bowen and Van Waes (2020), Eisenstein (2015), and Nottbusch (2010), keystroke analysis remains promising for asking sociolinguistic questions. For example, *how might the timing processes of enregistered and unmarked sociolinguistic variables differ*? *How might they differ for variables that are undergoing a change-in-progress through rapid diffusion across social networks*? *Would any such differences indicate how individuals and social networks recontextualize variables differently*? Consider the rapid diffusion of *(on) fleek* through Twitter in 2014 (see Grieve et al., 2017), which may be attributed to a viral video. An examination of the timing processes through which Twitter users composed *(on) fleek* in order to contribute to its rapid diffusion may provide insights into how this feature was adopted by users. For example, as suggested by keystroke analysis literature, would newly adopted linguistic features, those that are highly salient, or those undergoing a change to their indexical field of meaning, be composed more slowly or experience longer pauses before and after inputting? Keystroke analysis, in addition to analysis of the frequency of use and discursive structuring of these text-based features, could therefore illuminate how individuals are meaningfully taking part and contributing to language change.

Lastly, I suggest that in order to examine the wealth of text-based data that may be commonly diffused through social media, it is important to expand beyond computer terminals. After all, well over 80% of Twitter use may be conducted on mobile devices (WSJTech, 2014, Apr 14), and writing interfaces on mobile touchscreen devices involve qualitatively different input-processes from other writing interfaces (see Farman, 2012; Mangen, Anda, Oxborough and Brønnick, 2015; Parisi, 2018). Indeed, as suggested by Brock (2020), Twitter borrowed heavily from SMS architecture and interfaces, and therefore likely encouraged compositional habits similar to texting. Therefore, even though preliminary studies into writing processes for composing *simulated* tweets on computers has demonstrated value (see Leijten et al., 2012), observing the compositional processes of these registers as they occur on mobile devices may yield more “naturalistic” observations.

### Research Questions

Based on the above, this study therefore seeks to examine the following research questions based on the keystroke-logging data:

1. RQ1: What are the frequencies of occurrence of paralinguistic variables *Haha* and *Lol* in text messaging, and what is their distribution according to turn-taking structures in asynchronous messaging?
2. RQ2: What are the timing processes of *Haha* and *Lol* in text messaging, and how do these timing processes reflect turn-taking structures in asynchronous messaging?

As will be detailed in the following section, this study therefore applies keystroke-logging methods for writing on mobile devices, and further explores further means of asking sociolinguistic questions for text-based language that is common to written registers in mobile communication.

## Methods

The present study reports on keystroke-logging of mobile devices. This follow Eisenstein’s (2015) suggestion that keystroke-logging methods may more closely observe the production of text-based linguistic content in popular mobile platforms, as well as Schneier and Kudenov’s (2018) demonstration of how keystroke-logging data can be successfully collected from mobile devices. This study was therefore designed to observe how text is digitally articulated on mobile devices as individuals compose and send text-messages to members of their social network. This involved developing a mobile keyboard application for Android devices to log keystroke data, collecting keystroke data from participants (*N* = 10), and conducting pause-based analysis of keystroke data pertaining to paralinguistic features from seven of those participants. This section will provide further details on the designs of LogKey, how data was collected as part of this study, briefly overview data output and analysis, and discuss this study’s sample size.

### Designing LogKey

In the recent decade, writing scholars, particularly Van Waes et al., (2012) and, have made concerted efforts to establish standardized recommendations for designing keystroke loggers for computer-based writing. These recommendations outline use of XML-structure to log the sources of computer-based actions, such as input from a keyboard or mouse, as well as how to operationally (or even algorithmically) define a sequence of actions, such as how to define the temporal threshold of a *pause* during writing. While Van Waes et al. (2012) do discuss methods of how to accommodate other means of input, particularly through speech recognition, use of a stylus, or even use of the “swipe” action on a touchscreen device, these recommendations do not explicitly address how a keystroke on a computer keyboard with tactile keys is *not* the same as a keystroke on a virtual keyboard.

As found in Schneier and Kudenov (2018), the technological distinctions between a computer keyboard and a virtual keyboard have a significant impact for how to log keystrokes. Computer keyboards have keys with binary up or down depressions; while virtual keyboards have keys that *simulate* the up or down depression of a key according to how the electronic charge of a finger comes into contact with the corresponding image of a key on screen (Andre et al., 2005; Westerman and Elias, 2006). Van Waes et al. (2012) outline that the times of each down press of a key and release of a key should be measured in order to determine the time between one key’s release and the next key's depression, what is called the inter-key interval (IKI). On a touchscreen, though, how can we measure the IKI if there is no *depression* of a key but instead *contact* with the screen?

The method for logging keystrokes in this study therefore intended to accommodate the technological configuration of what it means to press a virtual key on a virtual keyboard, as well as improve upon the methods explored in Schneier and Kudenov (2018) wherein participants (*N* = 5) used a smartphone with a keystroke logger built directly into the functionality of this phone. The method for keystroke logging in this study therefore intended to, 1) allow participants to use their own personal mobile devices in order to observe them using devices they were presumably familiar with and comfortable using; 2) observe participants over a longer span of time in order document everyday compositional habits on their mobile devices; 3) log what applications individuals were using when keystrokes were logged; and 4) log the time between the initial press of the key that was pressed and the previous key. In regards to item 4, this method replicates Schneier and Kudenov’s (2018) operationalization of the IKI, which Plank (2016) suggested is most valuable. In order to address the above needs, a virtual keyboard application was designed and constructed for this study, an application which could be substituted for the standard virtual keyboard. This app, called LogKey, was designed for the Android mobile operating system^3^.

**FIGURE 1:**
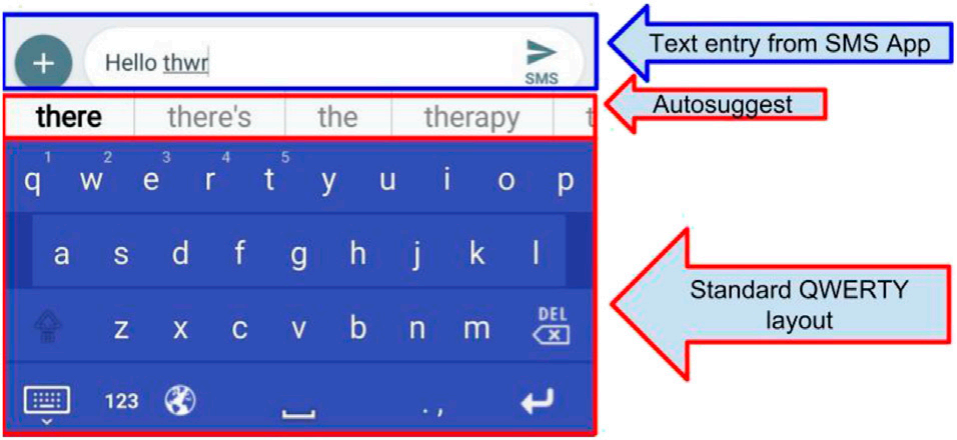
The LogKey keyboard, with autosuggest options.

The primary features of LogKey, from the perspective of users interacting with the Graphical User Interface (GUI), is that the application would appear as a standard QWERTY layout virtual keyboard, and include autosuggested text (see Figure 1). Like the standard Android keyboard, this virtual keyboard acts as a separate layer on top of whichever application is in use.

### Study Procedures

Participants (*N* = 10) were recruited through snowball sampling methods, primarily through various online message-boards and list-servs commonly accessed by undergraduate and graduate students at a large university in the mid-Atlantic region of the United States. Recruitment materials informed participants that the study was intended to learn about how individuals communicate through text-messaging, and would involve using an unreleased keyboard application for Android OS. Sociolinguistic studies of mobile communication, particularly texting, frequently involve university students (Thurlow and Brown, 2003; Baron and Ling, 2011; Spilioti, 2011; Androutsopoulos, 2014a) because they tend to be technologically verbose (Baym et al., 2004; Ledbetter, 2008; Lenhart, 2015) and may be seen as transitioning between youth- and adult-centered identity practices (Eckert, 2000). Further, Pew Research Center reports from 2014 further suggest that they (by the time of the target date of the study in 2018) are accustomed to predominantly using mobile smartphones (76% of 15–17 year-olds) for a range of purposes including general internet use, text-messaging, video chat, social media use, other messaging applications, and various other communicative applications (Lenhart, 2015).

Following completion of the Informed Consent process, participants were asked to complete two interviews (pre- and post-study), a brief observation session, and use the LogKey keyboard application for a period of seven days. The 5–10-min pre-study interview briefly addresses participants' habits and history using mobile communication media, which social ties they generally communicate with and how they describe those relationships (e.g., close friend, parent, roommate), as well as participants’ perceptions and attitudes regarding their mobile communicative practices within their interpersonal networks (Ledbetter and Mazer, 2014). Following the pre-study interview, participants were instructed how to download and install the LogKey application, as well as obtain Third-Party Consent through an Interlocutor Consent Script^4^ that was sent to individuals they expected to text regularly throughout the week, as well as any individuals who would text them later in the week. Obtaining third-party consent was deemed important because even though these third parties were not directly participating in the study (i.e., they were not using LogKey to have their keystroke data logged), the messages that they sent to participants and the messages that participants sent back to them (both of which were part of data collection) arguably *belonged to both* parties, and contained private information regarding both parties meaning these third parties had the ethical right to consent to participate indirectly or not.

At the conclusion of the seven days participants met again with the researcher. During this meeting the researcher instructed participants on how to securely transfer keystroke log-file data from the LogKey application to the researcher. Participants were also instructed how to download and install the SMS to Text application (SMeiTi, 2017), and to export textual log-file data from application to their SD card, and then transfer electronically to the researcher. The researcher then went through the log-file data with the participants in order to conduct a spot-check of the data, particularly to make sure that no data from third-parties who did not grant written permission to the participants be included in the data set. Following this, the researcher then conducted a brief post-study interview that discussed the participants’ experiences using the LogKey application throughout the week as well as what conversations they engaged in through their mobile devices.

### Keystroke Data Output

Adapting the recommendations from Van Waes et al. (2012), as well as lessons from Schneier and Kudenov (2018), keystrokes from use of LogKey were logged and stored in a comma-separated value (CSV) file format, as demonstrated in Table 1. This log file separates each log event into individual rows and is sorted chronologically, and each log event then has several corresponding values expressed in individual columns including: The date and time of the event (Date); The value of the log event as seen from the keyboard layout (Event Log); The category of the log event (Event Type); The Unix time in milliseconds of the initial press of the key (Time MS); The time elapsed between the initial press of the log event and the previous log event’s initial press (Pause MS); The application in use when the log event was recorded (Application). As can be seen in Table 1, this output can show when and what autosuggest options are presented to the user, as well as when and what autosuggest option they choose.

**TABLE 1:** Example of log file data in comma-separated value form.

Date	Event Log	Event Type	Time MS	Pause MS	Application
14:30:48	t	Key	1505154648754	553	Messaging
14:30:48	h	Key	1505154648918	68	Messaging
14:30:49	[The, that, this, they, there]	Auto_options	1505154649008	n/a	Messaging
14:30:49	e	Key	1505154649086	168	Messaging
14:30:49	SPACE	Key	1505154649679	549	Messaging
14:30:50	e	Key	1505154650478	696	Messaging
14:30:50	m	Key	1505154650630	55	Messaging
14:30:50	o	Key	1505154650866	128	Messaging
14:30:51	j	Key	1505154651241	281	Messaging
14:30:51	[Emoji, emojis, emojis, Emoji's]	Auto_options	1505154651306	n/a	Messaging
14:30:51	i	Key	1505154651422	153	Messaging

A disadvantage to the software configurations of mobile keyboard application on Android is that, as mentioned, only the simulated keys of the keyboard itself can be logged. This means that depending upon the application that the keyboard is being used with, such as the various SMS applications for Android, transmitting a message may not be recorded as a log event. In addition to not knowing when a text message is sent, LogKey is also unable to present a summative record of the message that was sent to interlocutors. The application SMS to Text, however, allowed participants to export SMS messages to the SD card on their personal mobile device. These exported messages may be saved in the form of a text (.txt) file or comma-separated value (.csv) file., and included information about the time of transmission, whether the message was sent to or from the participant, the number of the interlocutor, the name stored in the participant’s contacts list, and the textual content of the message sent or received. Together with the keystroke log-file data, as seen in Table 1, the text logs can be coordinated so that keystrokes may be corresponded to specific messages.

In merging and coordinating both data sources into a single matrix, it was possible to examine how a synchronous sequence of keystrokes constructed an asynchronous message that was transmitted from a participant's device. Further, using a combination of computational and manual coding, it was possible to demarcate individual word units as well as entire message units from a given sequence of keystroke activity. Using LogKey’s data output that displayed each sequential keystroke, the associated alphanumeric key or the SPACE key, and the IKI of that key, it was possible to identify series of sequential alphanumeric keys that may represent the intent to type a particular word. For example, an [H] key with an IKI above 2000 ms, followed by the keys [e], [l], [l], and [o] followed by the [SPACE] key likely represents a sequential burst of alphanumeric keystrokes activity from typing the word ‘Hello’ that I term *keybursts* or KBs. A series of embedded If-Then conditional formulas in MS Excel then labeled the first alphanumeric key in a keyburst (i.e., the inter-KBs, or the pause before the keyburst) that occurred immediately after non-alphanumeric keys or keybursts over 2000 ms, as well as the following alphanumeric keys in the keyburst up until a non-alphanumeric key (i.e., the intra-KBs). Doing so made it possible to group all keys within a keyburst and to distinguish the timing processes of the first key in a keyburst from the others (e.g., the [H] would be labeled “inter-key” and the keys [e], [l], [l], and [o] each labeled ‘intra-key’). Future versions of LogKey may designed to computationally produce this data as log-files are compiled, rather than through post-hoc tagging.

As discussed above, chunking keystroke activity into bursts, such as *pause* or *revision bursts*, is a common practice in keystroke analysis in order to infer linkages between linguistic, writing, and cognitive processes (Leijten et al., 2015; Galbraith and Baaijen, 2019). Further, boundaries for word units are often distinguished by use of function keys, i.e., the space bar or keys for punctuation (Van Waes et al., 2015). In other words, analysis of keyburst units will be similar in form and function to identifying potential word units according to keystroke pauses tagged as “BEFORE WORD” or “AFTER WORD” in *InputLog* (Leijten and Van Waes, 2013; Van Waes et al., 2015).

Identifying sequences of keystrokes in such a manner is a highly productive method for operationalizing input-based activity with purposeful communicative practices (see Van Waes et al., 2012); albeit, it does make at least two assumptions. The first assumptions is that a given sequence of alphanumeric keystroke logs reflect a participant intentionally composing a particular linguistic unit. While this may seem rather matter-of-fact, as Suchman (1987) has cautioned, human input and interaction with machinic interfaces (i.e., a log file record) reflects the machine’s designs and constraints as much as the human user’s intentions. In other words, at the same time a given burst of keystrokes may reflect a participant intentionally writing a particular word, it may also reflect unintentional keypresses (i.e., a pocket-dial or pressing an errant key). A second assumption is that identifying keybursts, particularly the timing processes of these keybursts, will provide important evidence of psychomotor processes involved in word recall and procedurally inputting that word through QWERTY keyboard. Writing process research has suggested that the time between the first key inputted in a burst of keystroke activity or between typing separate words evidences such cognitive processes (Miller, 2006; Hayes, 2012; Leijten and Van Waes, 2013; Leijten et al., 2014; Mangen et al., 2015). This study will therefore focus largely on the time before a keyburst was inputted, i.e., the IKI value of the inter-KB key, and the average time required to input all subsequent keys in the keyburst, i.e., the average IKI value of the intra-KB keys in a keyburst.

### Linguistic Analysis of Keystroke Data

In order to investigate the research questions, this study first identified all variants of *Haha* or *Lol* that occurred in 908 sent text-messages that participants procured for this study. This involved creating a matrix that identified each token, the message it occurred in, its turn-taking position (i.e., initial, medial, or terminal), and, by coordinating with the keystroke data, the inter-KB and intra-KB values. It should also be noted that the turn-taking position was determined by the position of the variable within the transmitted text-message, not within the sequential process of composing the message, and that the code for the initial position included instances in which a transmitted message only contained one of these variables.

Further, using the dplyr^5^ package in R Studio, this study generated a matrix with every unique keyburst produced by all participants, including the number of times each keyburst occurred, and its relative frequency among all keybursts. Using this matrix, it was possible to hand-code all unique variants of *Haha* and *Lol* (see Tables 2 and 3), and then identify every occurrence of *Haha* and *Lol* throughout the keystroke data (texting and all) as well the application in use when each token was composed*.* Due to the manner in which keyburst boundaries were determined (see *Section 3.3*), variants necessarily included keybursts that were never transmitted, likely a result of repairs related to typing errors. For example, as shown in Table 3, the list of variants of *Haha* include readily apparent variants such as *haha* and *hahah,* as well as unapparent variants such as *hwhw* and *jehe*. These two variants, which occurred only once each, were necessarily identified as variants of *Haha* because they were deleted and replaced with more recognizable *haha* or *hehe*, and because the repaired characters are adjacent to the mistyped characters on the QWERTY keyboard. I include such keybursts as variants in this list because, even though they are clearly not spelled the same as the more iconic and frequent variants and are most likely *typos*, these keybursts likely reflect habitualized articulatory processes the same as the others.

**TABLE 2:** the top 75 most frequent keybursts in texting data.

rank	Keyburst	Absolute frequency	Relative frequency	Characters
1	i	334	3.56%	1
2	To	224	2.39%	2
3	The	167	1.78%	3
4	You	139	1.48%	3
5	And	122	1.30%	3
6	Im	121	1.29%	2
7	a	119	1.27%	1
8	t	103	1.10%	1
9	is	90	0.96%	2
10	It	78	0.83%	2
11	me	73	0.78%	2
12	So	73	0.78%	2
13	That	69	0.74%	4
14	do	68	0.73%	2
15	my	65	0.69%	2
16	u	63	0.67%	1
17	e	61	0.65%	1
18	No	59	0.63%	2
19	s	59	0.63%	1
20	But	56	0.60%	3
21	y	54	0.58%	1
22	Have	53	0.57%	4
23	we	51	0.54%	2
24	d	50	0.53%	1
25	Of	50	0.53%	2
26	Just	49	0.52%	4
27	are	48	0.51%	3
28	For	48	0.51%	3
29	In	48	0.51%	2
30	o	48	0.51%	1
31	be	43	0.46%	2
32	n	43	0.46%	1
33	w	43	0.46%	1
34	At	42	0.45%	2
35	Good	38	0.41%	4
36	Oh	38	0.41%	2
37	On	38	0.41%	2
38	g	37	0.39%	1
39	Its	36	0.38%	3
40	Or	36	0.38%	2
41	was	36	0.38%	3
42	What	36	0.38%	4
43	All	35	0.37%	3
44	Dont	35	0.37%	4
45	How	35	0.37%	3
46	I	35	0.37%	1
47	not	35	0.37%	3
48	If	34	0.36%	2
49	m	32	0.34%	1
50	r	31	0.33%	1
51	Go	29	0.31%	2
52	h	29	0.31%	1
53	Get	28	0.30%	3
54	Will	28	0.30%	4
55	Yeah	28	0.30%	4
56	Your	28	0.30%	4
57	This	27	0.29%	4
58	Up	27	0.29%	2
59	can	26	0.28%	3
60	Lol	26	0.28%	3
61	Okay	26	0.28%	4
62	f	25	0.27%	1
63	k	25	0.27%	1
64	Like	24	0.26%	4
65	Hey	23	0.25%	3
66	l	23	0.25%	1
67	Want	23	0.25%	4
68	Know	22	0.23%	4
69	Love	22	0.23%	4
70	An	21	0.22%	2
71	b	21	0.22%	1
72	he	21	0.22%	2
73	Time	21	0.22%	4
74	Haha	20	0.21%	4
75	Now	20	0.21%	3

**TABLE 3:** Frequency and occurrence of all Haha or Lol Keybursts from Text-Messaging Data.

Variable	Variant	Occurrrence	Frequency of texting keybursts
Lol	Lol	26	0.2773%
Lol	Lloll	1	0.0107%
Lol	Lol	1	0.0107%
Lol	Lolim	1	0.0107%
Lol	Lolol	1	0.0107%
Lol	Lolthsy	1	0.0107%
	**Total lol keybursts**	**31**	**0.3307%**
Laughter	Haha	20	0.2133%
Laughter	ha	4	0.0427%
Laughter	Hah	2	0.0213%
Laughter	Hahahahaha	2	0.0,213%
Laughter	Ehe	1	0.0107%
Laughter	Ehehehe	1	0.0107%
Laughter	Haah	1	0.0107%
Laughter	Hahah	1	0.0107%
Laughter	Hahah	1	0.0107%
Laughter	HAHAH	1	0.0107%
Laughter	Hahaha	1	0.0107%
Laughter	Hahahah	1	0.0107%
Laughter	Hahahaha	1	0.0107%
Laughter	Hahahahahha	1	0.0107%
Laughter	Hahahahhh	1	0.0107%
Laughter	Hahahit	1	0.0107%
Laughter	Hwhw	1	0.0107%
Laughter	Jehe	1	0.0107%
	**Total laughter**	**42**	**0.4481%**

### A Note About Participant Pool Size

Since this study included only 10 participants, its findings are not necessarily generalizable. Nevertheless, I wish to put this small sample size in the context of writing process research. First, as discussed above, keystroke-logging studies may often involve a small number of participants. For example, Leijten et al. (2014) involved *N =* 1, Leijten et al. (2015) involved *N* = 2, and Bowen and Van Waes involved an *N* = 1. In such studies, researchers are less concerned with generalizing about entire populations and more so focused on examining writers in-depth and in-context in order to challenge and build upon theoretical models of writing (see Leijten et al., 2014). Second, in spite of the small participant pool, this study observed individuals writing for longer durations and as part of everyday mobile communication habits. This compares drastically to common keystroke studies that writing tasks of shorter duration in formal settings. For example, participants in Leijten et al. (2012) composed short simulated tweets; participants in Leijten et al. (2015) composed texts typically written in under 10 min; participants in Van Waes et al. (2010) revised short sentences; and participants in Nottbusch (2010) composed short sentences in response to stimuli. Even in studies that observed writers composing formal reports over multiple days, such as Leijten et al. (2014) and Bowen and Van Waes (2020), participants engaged in individual writing episodes that would last between 20 min to several hours. In other words, a study of writing on mobile devices, which may involve shorter forms of writing, may nevertheless yield similar data sets as Leijten et al. (2014) and Bowen and Van Waes (2020), in addition to involving writing more frequently throughout a participant’s everyday life.

## Results

This section will provide an overview of the keystroke data collected from this study. I will start with more descriptive summary of the data collected from participants’ use of the LogKey keyboard across all applications, and then narrow in further by discussing the occurrence of *Haha* and *Lol* in the keystroke data in order to address RQ1, and the timing processes of those features in order to address RQ2.

### General Overview

**FIGURE 2:**
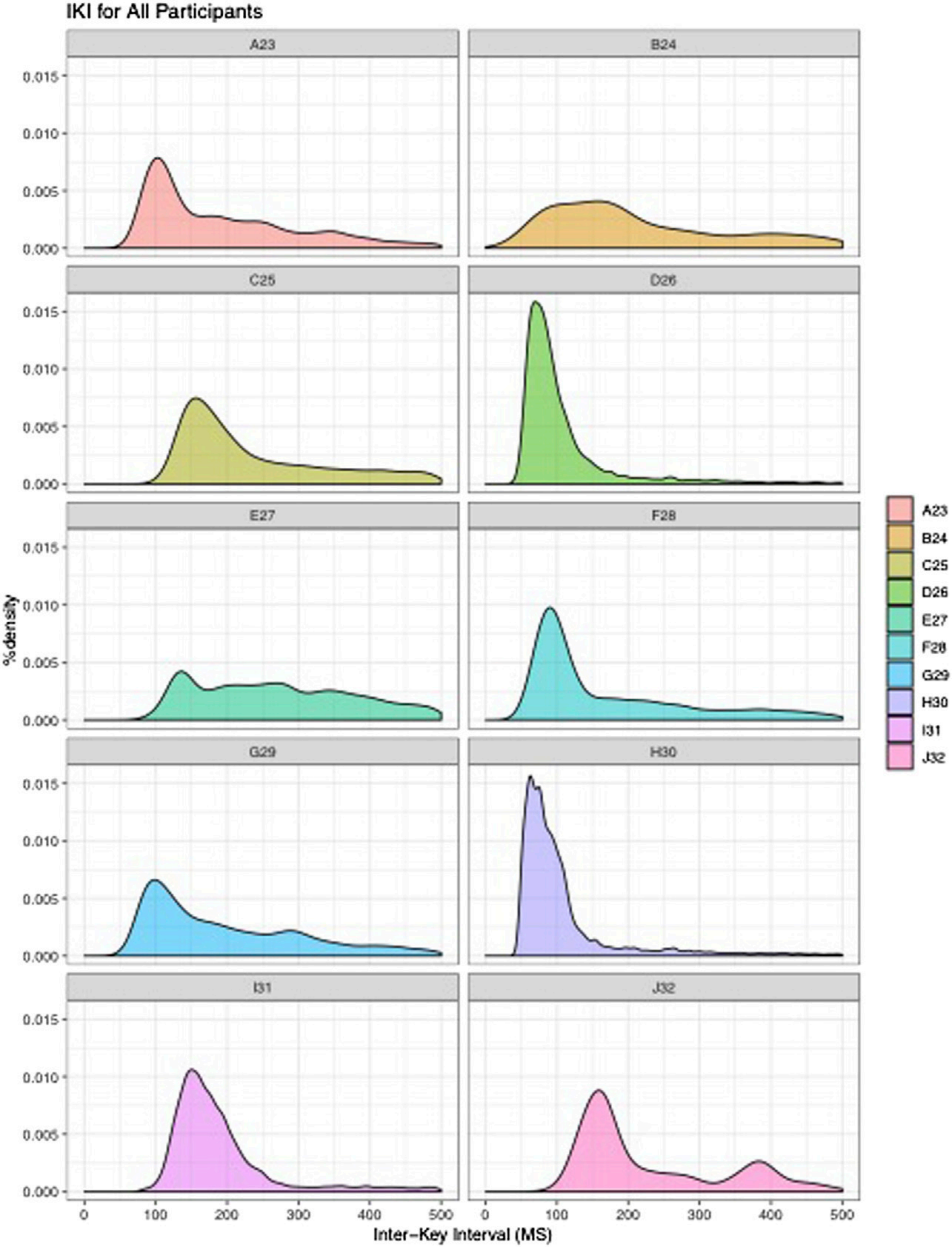
Density plots showing IKI for all participants.

The participants in this study (*N* = 10) reflect the targeted population, in that they all used mobile phones running Android OS as their personal devices, were between the ages of 18–35, and were all college-educated. A majority of the participants were currently enrolled in undergraduate studies at a four-year institution (*N* = 7), while the remaining participants were either enrolled in graduate studies (*N* = 2) or were working professionals (*N* = 1). All participants reported that text-messaging was among their most-used communication or messaging applications, although apps such as Snapchat, Instagram, GroupMe, WhatsApp, Facebook, Facebook Messenger, Twitter, and email were frequently used as well. In total, 179,996 keystrokes, or 32,274 keybursts (see Table 4) were collected from all 10 participants’ use of LogKey, with the fewest number of keybursts produced by Participant B (*N* = 152), and the most produced by Participant H (*N =* 6,353). All participants varied from one another, although each participant displayed a general tendency for typical IKIs to cluster below 500 ms (see Figure 2). Nevertheless, it is worth noting that, with the exception of participant B, who noted frequent frustration with the LogKey keyboard and produced the least amount of data, and participant E, who was the oldest participant, that participants displayed dense patterning of their IKIs wherein each appeared to typically type at a speed within a range of 100 ms.

**TABLE 4:** General summary of keybursts for all participants.

Participants	Total keybursts	Total keybursts >1 keystroke	Median of inter-KB IKI	Median of average intra-KB IKI	Median of intra-KB IKI average (≥3 characters)	Median of intra-KB IKI average (<3 characters)
A	4,585	3,727	184	96	65.0	144
B	152	127	492	110.95	76.33	154.0
C	4,311	3,652	216	148.8	98.0	221.22
D	1,223	1,107	106	62	47	71.60
E	4,375	3,731	369	206.4	146.0	275.80
F	2,121	1786	216	86.0	52.00	151.5
G	3,119	2,716	229	111.1	78.3	158.5
H	6,353	5,377	104	54.0	41.0	67.80
I	5,071	4,381	193	117.5	95.0	140.80
J	964	863	183	171.6	99.0	217.0
**Total**	**32,274**	**27,467**	**201**	**103**	**72**	**149**

**FIGURE 3:**
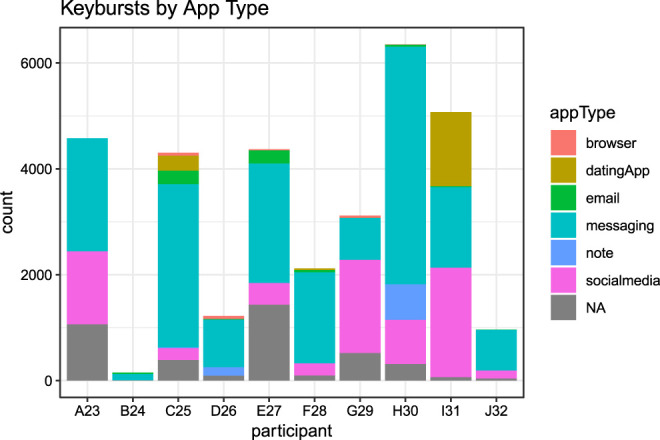
Keyburst count for Application Type for each participant.

Participants additionally used a variety of applications during their participation, which confirmed self-reports from pre- and post-study interviews about the applications they use within a typical week. Figure 3 below shows that while participants varied slightly in the degree to which they produced text through different applications, overall keybursts were primarily produced in messaging (55% of the total keybursts), followed by social media apps (21.8% of total keybursts). Furthermore, when examining the IKI of the first key in a keyburst produced in a given application (i.e., the inter-KB value), keybursts produced in messaging applications appeared to significantly predict lower inter-KB values when compared to keybursts in dating apps, email, note-taking apps, and browsers (see Table 5). Interestingly, messaging and social media applications did not appear to differ significantly from one another.

**TABLE 5:** Linear Mixed Effects Models (with participant as random intercept) examining inter-KB and average intra-KB of keybursts across application type.

Predictors	Inter -KB		
	Estimates	CI	*p*
(Intercept)	200.72	164.59–236.84	<0.001
Browser (compared to messaging)	37.39	20.10–54.68	<0.001
Dating apps (compared to messaging)	−15.07	−20.98–−9.15	<0.001
Email (compared to messaging)	−30.07	−38.71–−21.44	<0.001
Note (compared to messaging)	14.29	6.81–21.77	<0.001
Social media (compared to messaging)	0.20	−3.14–3.54	0.906
Random effects			
σ^2^	8,794.01		
τ_00_	3,379.95 _participant_		
ICC	0.2,776,377		
Observations	22,330		
Marginal *R* ^2^/Conditional *R* ^2^	0.004/0.280		

Of the 32,274 total keybursts produced by participants, 8,290 were unique keybursts, and 5,908 of those unique keybursts occurring only once. In other words, keybursts that occurred only once, such as *lolol*, make up a total of 18% of all keybursts produced in the study. The most frequently occurring keybursts were common stop words such *to*, *the*, *a,* and, the first- and second-person subject pronouns ranked first and fourth, respectively. Furthermore, the most frequently occurring variants of *Haha* and *Lol* (i.e., *haha* and *lol*) represented the 43rd and 61st most frequently occurring keybursts, respectively (See Table 2).

### Frequency of *Haha* and *Lol*

As stated in the RQ1, this study sought to examine the frequency of occurrence of two paralinguistic variables, *Haha* and *Lol*. As previously mentioned, eight participants procured 908 individual transmitted messages containing 7,329 words for this study. This number includes 62 messages sent over WhatsApp or Google Chat by participant B and J, respectively (due to how they configured the texting settings on their phones), and excludes messages transmitted using MMS, Advanced Messaging, or messages that participants sent to third-parties who did not consent to participate in the study. Hand-coding of the 908 messages identified 32 tokens of *Haha* and 26 tokens of *Lol*, meaning that *Haha*’s relative frequency among all transmitted messages was 0.44% while *Lol*’s relative frequency was 0.35%. It is noteworthy that these relative frequencies are roughly similar to those found in Tagliamonte and Denis (2008) and Tagliamonte, (2016), although, as Table 6 shows, the relative frequencies of *Haha* and *Lol* varied among individual participants. For example, *lol* made up 1.22% of all words participant J transmitted, and *haha* made up 0.79% of all words participant C transmitted. Further, three out of the six participants who transmitted either of these variables categorically transmitted one or the other.

**TABLE 6:** Frequencies of *Haha* and *Lol* by participants in sent texting data.

Participant	Haha (absolute/relative frequency)	Lol (absolute/relative frequency)	Total messages sent	Total words in messages
A	2/1.27%	1/0.63%	26	157
B	—	—	1	4
C	14/0.79%	10/0.56%	280	1769
D	—	—	30	416
F	2/0.16%	2/0.16%	104	1,234
H	14/0.62%	—	255	2,255
I	—	8/0.74%	151	1,086
J	—	5/1.22%	61	408
**Total**	**32/0.44%**	**26/0.35%**	**908**	**7,329**

As demonstrated in Meredith and Stokoe (2014), not all content composed for text-based messaging is sent. This study therefore sought to examine the frequencies of keybursts composed by participants as part of texting, regardless of whether those keybursts were transmitted or not. In total, 9,364 keybursts were composed by participants as part of constructing the 908 transmitted messages. This potentially means that, when comparing with the 7,329 words that were sent, that potentially 2,047 keybursts were composed *but deleted* by participants. A frequency matrix of the texting keybursts identified 3,009 unique keyburst as well as a counts of the absolute frequencies and calculations of the relative frequencies. The top 75 most frequent keybursts are shown in Table 2, which includes the variants *lol* and *haha* as the 60th and 74th most frequently occurring keybursts. Hand-coding of this matrix identified 18 variants of *Haha* and six variants of *Lol*, as shown in Table 3, which includes variants like *lolol* and *hahahahhh*. In total, this identified 42 variants of *Haha* (10 more than the transmitted tokens), or a relative frequency of 0.45% of texting keybursts, and 31 variants of *Lol* (5 more than the transmitted tokens)*,* or a relative frequency of 0.33% of texting keybursts. This means that, while 10 tokens of *Haha* and five tokens of *Lol* were composed but never transmitted, their relative frequency is consistent across keyburst and transmitted data. Further, as shown in Table 7, participants varied with regard to how frequently they composed either variable, and, interestingly, this examination revealed that participant D indeed composed both *Haha* and *Lol* in spite of never sending either in a text message.

**TABLE 7:** Frequencies of *Haha* and *Lol* keybursts in texting data.

Participant	Haha (absolute/relative frequency)	Lol (absolute/relative frequency)	Total keybursts from Texting Data
A	2/0.97%	1/0.48%	207
B	—	—	4
C	17/0.75%	10/0.44%	2,281
D	1/0.23%	2/0.46%	430
F	3/0.26%	2/0.17%	1,154
H	19/0.57%	—	3,312
I	—	10/0.70%	1,437
J	—	6/1.11%	539
**Total**	**42/0.45%**	**31/0.33%**	**9,364**

It is also important to note that both *Haha* and *Lol* were composed by participants in other applications besides texting. When examining the entire data set collected from LogKey, which includes keystrokes and keybursts from communicative and non-communicative applications, keybursts such as *lol* and *haha* maintain a similar level of relative frequency; occurrences of *lol* and *haha* keybursts (including their variants) therefore respectively represent 0.45% and 0.40% of all keybursts collected from this study.

#### Turn-Taking Structures

As discussed in *Section 2.2*, paralinguistic features are commonly deployed at turn-taking positions in asynchronous messaging, which was verified by this study. Across participants, both *Haha* and *Lol* were predominantly used in the initial or terminal positions of a sent message. 23 tokens of *Haha* occurred in the initial position, seven in the terminal position, and two at a medial position between clause boundaries. 10 tokens of *Lol* occurred in the initial position, 13 in the terminal position, and three at a medial position between clause boundaries. While this study does not necessarily argue that *haha* and *lol* are variants of the same variable, a chi-square test of independence was performed to examine the relation between use of *haha* or *lol* at the initial or terminal position in a message. The relation between these was significant, X^2^ (1, *N* = 53) = 6.1013, *p* = 0.0134, as *Haha* was more likely to be in the initial positions. As is shown in Table 8, individual participants varied in their use of these variables at the different positions. For example, Participant H nearly exclusively used *haha* in the initial position, and Participant I similarly tended to use *lol* in the terminal position, which suggests that the above chi-square test may have been biased by the observed habits of individual participants. The situational use of these features at these turn-taking positions will be further discussed in *Section 5*.

**TABLE 8:** *Haha* and *Lol* by turn-taking position.

Participant	Haha initial – terminal – medial			Lol initial – terminal – medial		
A		2		1		
C	9	4	1	3	5	2
F	1	1		2		
H	13		1			
I				1	6	1
J				3	2	
**Total**	**23**	**7**	**2**	**10**	**13**	**3**

### Timing Processing of Composing *Haha* and *Lol*

As demonstrated in Tables 4 and 5, participants’ articulatory timing processes varied, as would be expected from keystroke data. Indeed, while similar variations were found when examining participants’ individual timing processes for composing *haha* and *lol* in texting data, in general participants tended to take more time to compose *haha* and *lol* than typical keybursts. As shown in Table 9 below, across seven participants who composed *haha* or *lol* in the texting data, the median intra-KB average (i.e. the average speed of composing a keyburst) was 85ms, whereas *Haha* was 135.38 ms and *Lol* 176.6 ms.

**TABLE 9:** Median intra-KB averages of *Haha* and *Lol* keybursts

Participant	Haha Tokens	Haha Median intra-KB Average (ms)	Lol Tokens	Lol Median intra-KB Average (ms)	Median intra-KB Average (ms) All Keybursts	Total Keybursts from Texting Data
*A*	2	336.0	1	74	103.75	207
*C*	17	212.2	10	248.7	159.2	2,281
*D*	1	62	2	67.97	59.25	430
*F*	3	210.4	2	233.5	84.33	1,154
*H*	19	71.00	—	—	52	3,312
*I*	—	—	10	123.8	114	1,437
*J*	—	—	6	196.7	176.3	539
*Total*	**42**	**135.38**	**31**	**176.6**	**85**	**9,364**

That *Haha* and *Lol* keybursts appeared to have different timing patterns was confirmed when examining the inter-KB values (i.e., the time prior to composing the keyburst). A linear mixed effects model (see Table 10) showed that variants of *Haha* and *Lol* keybursts were more likely than all other keybursts to have higher inter-KB values (*p* = 0.056 and *p* = 0.017, respectively). A possible interpretation of this is that participants took more time to cognitively select these specific features and then more time to compose them because they were dedicating more cognitive attention to use of these symbolic features, especially when compared to all other keybursts.

**TABLE 10:** Linear Mixed Effects Model for inter-KB and Haha or Lol keybursts (participant as random intercept) in texting data (excluding keybursts with inter-KB > 2000ms).

inter Total			
Predictors	Estimates	CI	p
(Intercept)	266.04	216.67–315.41	**<0.001**
Laughter	56.50	−1.48–114.48	0.056
Lol	57.51	10.34–104.68	**0.017**
Random effects			
σ^2^	66,222.53		
τ_00_ _participant_	4,764.82		
ICC _participant_	0.07		
Observations	8,687		
Marginal *R* ^2^/Conditional *R* ^2^	0.001/0.068		

**FIGURE 4:**
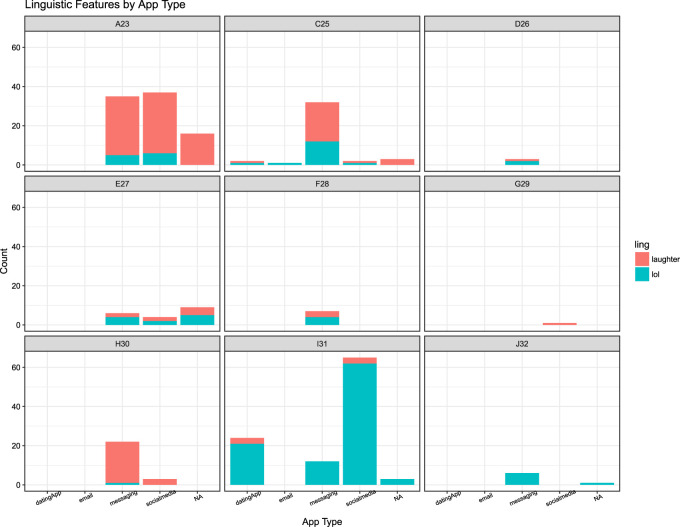
Occurrence of Haha or Lol feature by application type for each participant.

This interpretation is further evidenced by the temporal data concerning *Haha* and *Lol* keybursts beyond texting data. As shown in Figure 4, participants composed *Haha* and *Lol* in texting and all other mobile applications during their participations, totaling 145 *Haha*s and 149 *lol*s across all keystroke data. However, welch two-sample t-tests indicated there was no significant difference between inter-KB values for either *Haha*s composed in messaging and non-messaging apps (*t* [83.224] = 0.16123, *p* = 0.8723) or *Lol*s in messaging and non-messaging apps (*t* [76.157] = 1.2898, *p* = 0.201). A possible interpretation of this is that these features are used similarly across other mobile writing registers, or that the other mobile apps that participants composed these features in bear resemblance to texting. Indeed, besides messaging apps, these features were composed in dating apps (4 *Haha*s, 22 *Lol*s) and social media apps (41 *Haha*s, 71 *Lol*s), which may share similar asynchronous messaging structures.

#### Turn-Taking Structures and Timing

As discussed in *Section 4.2.1*, participants overwhelmingly composed *Haha* and *Lol* keybursts in the initial or terminal positions of text-messages as opposed to medial positions within messages, and *Haha* was more likely to be used in the initial position. Interestingly, the timing of *Haha* and *Lol* appeared to differ at different turn-taking positions, as *Haha*s and *Lol*s in the initial position were generally composed faster than in the terminal position. As shown in Table 11, this is most dramatic for *Haha* keybursts, where the median intra-KB average was over 200 ms *slower* in the terminal position than the initial or medial positions. While in general any keyburst in the initial position was composed faster than the terminal position (103.5 ms vs. 125.5 ms), when excluding keybursts with inter-KB values above 2000 ms, a linear mixed effects model did not appear to show any significant relationship between message position and intra-KB average across all participants (*p* > 0.05). A possible interpretation of this is that the psychomotor processes for composing the first and last words in a text message may involve greater cognitive demands, especially when compared to the general speed of composing medial keybursts, which would bear some similarity to speech rates toward the end of utterances (Sóskuthy and Hay, 2017).

**TABLE 11:** Median intra-KB averages of *Haha* and *Lol* by turn-taking position.

Position	Haha Tokens	Haha Median intra-KB Average (ms)	Lol Tokens	Lol Median intra-KB Average (ms)	All Keybursts Median intra-KB Average (ms)
Initial	23	121.00	10	174.6	103.5
Terminal	7	328.5	13	185.0	125.5
Medial	2	107.98	3	135.67	80.25
**Total**	**32**	**143.9**	**26**	**174.6**	**85.0**

**FIGURE 5:**
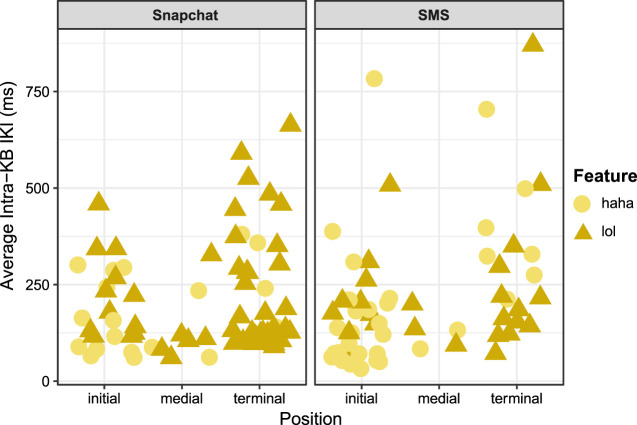
Jitter plot showing average speed of Lol and Haha for SMS and Snapchat data.

In order to further investigate the relationship between composition of these paralinguistic features at different turn-taking positions, additional tokens were included from tokens of *Haha* and *Lol* composed while the social media app Snapchat. Snapchat is a social media app that involves both photo-sharing as well as instant-messaging (Ekman, 2015; Jeong and Lee, 2017; Ilbury, 2018). As previously mentioned, six participants used Snapchat during their participation in the study, producing a total of 41 *Haha*s and 71 *Lol*s. When examining the strings of keystroke and keyburst data from the contexts in which these tokens occurred, they indeed appeared to resemble discrete messages. While Snapchat messages disappear after viewing, making it impossible to know who the participants were communicating with or even what the transmitted message was, it was possible to identify 74 *likely* messages from keystroke data. This included 19 *Haha*s and 55 *Lol*s. As shown in Table 12, a linear mixed effects model suggests that, when aggregating both variables together across texting and Snapchat messages, *Haha* or *Lol* in the terminal position was a significant predictor of higher intra-KB averages. Figure 5 visualizes this distinction as well. A possible explanation of this is that composing salient, paralinguistic features such as *haha* or *lol* at the end of a message on a mobile device, regardless of the specific register, may involve different psychomotor processes related to how participants were cognitively attending to how that paralinguistic cue functioned in context to their ongoing discourse. As will be further discussed in the following section, I argue that Brown and Levinson’s (1987) politeness framework offers one possible interpretation for this apparent distinction in digital articulatory processes.

**TABLE 12:** Linear mixed-effects model of Lol and Haha in Snapchat and SMS data (participant as random intercept).

Average Intra-KB IKI			
Predictors	Estimates	CI	*p*
(Intercept)	214.59	130.35–298.83	**0.001**
Medial (vs. Initial)	−60.41	−144.66–23.85	0.162
Terminal (vs. Initial)	71.58	13.12–130.04	**0.018**
Texting (vs. Snap)	−25.98	−99.50–47.54	0.491
Random effects			
σ2	19,674.96		
τ00 participant	5,800.19		
ICC participant	0.23		
Observations	132		
Marginal R2/Conditional R2	0.086/0.294		

## Analysis: Keystrokes in Context

In the above *Section 1* provided an overview of the keystroke data collected from this study, which identified the frequencies and timing processes of the paralinguistic features *Haha* and *Lol* that participants composed while text-messaging, as well as how these features functioned as part of turn-taking structures in messaging. Importantly, this analysis found that *Haha* and *Lol* were predominantly composed at the start or end of a message, and that they were composed more quickly at the start of a message when compared to the end. In this Section I will demonstrate how, as Van Waes et al. (2015) suggested, keystroke analysis may be combined with qualitative discursive analysis in order to better understand the interactional contexts through which individuals engage in text-based mobile communication. This will involve examining the interactional contexts in which participants composed *Haha* or *Lol* through the lens of Brown and Levinson’s (1987) politeness framework. In doing so, I hope to offer an approach that serves as one possible method to heed Nguyen et al.’s (2016) challenge to reconcile the analysis of macro-scale data channels obtained from computational methods (i.e., keystroke data) and discursive analysis of real-world social performances (i.e., sent text message data).

As discussed in *Section 2.2*, according to Brown and Levinson (1987), individuals may use various politeness strategies during communication, such as *shared laughter* (Jefferson, 1979) in order to mitigate possible positive or negative FTAs to themselves and interlocutors. The paralinguistic features *Haha* or *Lol* may function similarly to *shared laughter* by being deployed at turn-taking positions in messaging as a means of managing politeness strategies within asynchronous text-based mobile registers like text-messaging. I further suggest that this frames keystroke data, which provides insights into temporal dimensions of digitally articulation, as reflecting attention-paid-to-text, or how individuals may strategically manage composed and sent message content to negotiate their relationships through texting. I will attempt to demonstrate this through example conversations from participant data, particularly from participants C and H.

Participant C, who produced 17 *Haha* tokens and 10 *Lol* tokens, routinely composed these features faster at the start of a message than at the end. When examining how C used many of these tokens in context, they offer vivid examples of how these features strategically management politeness, particularly when in conversation with their partner. In Example C01, C and their partner are discussing beverages to purchase for a social gathering they are planning. In turn 01, C's partner references a specific alcoholic beverage in what appears to be a joke (as indicated by *Lmao* and the hot face emoji at the initial and terminal turn-taking positions), and C's response with an initial *Haha*, “Haha oh lordy,” in turn 02 therefore likely serves to indicate humorous reception and preserve their partner’s positive face. This is further supported by the fact that the intra-KB average of this *Haha* was 181.5 ms.

In Example C02, while discussing the logistics of coordinating a future trip, C’s partner declines C's suggestion in turn 02 (“That sounds like a process though lol”). In the next turn, C’s partner offers a joke, perhaps to mitigate their FTA from the previous turn. C’s response in turn 04 (“Its already a process”), which used some of their partner’s language from turn 02, appears to reject the joke, yet still includes a *haha* in the terminal position, composed with an intra-KB average of 328.5 ms. This terminal *haha* therefore appears to mitigate the FTA of rejecting their partner’s joke, and the slower composition time, when compared to the initial *Haha* in Example C01, may indicate that C deliberately attended to the content of their message in order to preserve their partner’s positive face. In other words, the faster speed of composing the initial *Haha* may be indicative of C reacting to their partner’s previous turn, but the slower speed of composing the terminal *haha* may indicate C was paying close attention to the message they were in the act of composing.

**Table:** 

Example C01			
Turn	Interlocutor	Time sent	Message
01	Partner	12:43:04	Lmao well I already have my pear cider and pink lemonade [HOT FACE EMOJI]
02	C	12:43:19	Haha oh lordy
03	Partner	12:43:44	Oh it's going to be sensational

Across these as well as other examples, participant C appears to use initial and terminal paralinguistic features for different strategies as part of turn-taking structures. In another exchange with their partner, the terminal *haha* in the message “Shes important to my mom, but not so much me haha” was composed at an average intra-KB IKI of 498.25 ms. Similarly, the *lol* in “Lol nvm haha” was composed at 172.6 ms (and the *haha* in the terminal position was 212.25 ms), whereas in “Mom what lol” it was composed at 297.3 ms. A possible explanation for taking more time to compose these features in the terminal position may be that the composer of the message needs to review the content of their message and mitigate any positive face threats to the receiver. In other words, composing *Haha* at the initial position may be cognitively retrieved and physiologically inputted faster than at the terminal position *because* they serve alternative discursive functions. For example, if a *Haha* at the initial position may be managing the positive face of the interlocutor, perhaps in reaction to the interlocutor offering a joke or sacrificing their own face (as in the message “Haha oh lordy”), then the faster speed may be indicative of the compulsion to quickly react to and placate the positive face of the interlocutor. In other words, composing these speeds at different discursive positions may correspond with face management strategies and even how the sender is paying attention to how their message content. This would bear resemblance to shared laughter’s function to coordinate the start of a responder’s turn and their acceptance of appropriateness (Jefferson, 1979), as well as Brown and Levinson’s (1987) observation that delayed responses in spoken turn-taking structures may signal a face threat based on prior turns.

Examining the keystroke data through the lens of politeness strategies also offers an explanation of revision patterns. As Meredith and Stokoe (2014) suggested, repairs in message construction, which are seen only by the individual composing the text, do more than correct textual errors—they may reflect how individuals are deliberately managing their interpersonal relationships. This may be demonstrated through participant H’s individual compositional habits, who was the fastest writer among participants with a median intra-KB average speed of 52 ms. H generally used variants of *Haha* at or even below this value, and predominantly in the initial position. As show in Example H01, the initial *Haha* in turn 04 (“Haha all right so should i just wear workout clothes?”) was composed at a rate of 50.5 ms, whereas in Example H02 the initial *Haha* in turn 04 (“Haha yea kinda i just have no idea whos coming or when but well see how it goes!!!”) was written at a rate of 149.25 ms. H wrote each of these messages to the same interlocutor, identified as Friend11, and after closer examination of the content and context of their messages suggests that the nature of their relationship changed during H’s week of participation in the study. For the first two days of H’s participation, as shown in Example H01, H and Friend11 primarily coordinated social activities, what Thurlow and Brown (2003) termed the *social-arrangement* orientation. However, on the third day of participation, Friend11 appeared to have left on a family vacation, so the following messages, as shown in Example H02, concerned catching up on their daily activities at a distance, what Thurlow and Brown (2003) termed the *informational-relational* orientation. The change in the speeds at which H composed initial *Haha*s to Friend11 may reflect a shift in communication orientations, which required H to respond to turns from Friend11 with message content that would maintain their relationship. This orientation likely would have afforded H more time to respond to messages, since they weren't time sensitive in the way the social-arrangement orientation was, and is evidenced by the fact that more time elapses between turns in Example H02 than in H01.

**Table:** 

Example C02			
Turn	Interlocutor	Time sent	Message
Turn	Interlocutor	Time sent	Message content
01	C	8:52:27	We can always do the week-of ticketd
02	Partner	8:53:05	That sounds like a process though lol
03	Partner	8:53:16	Like every week we have to see if we have to go to cleveland that week
04	C	8:53:25	Its already a process haha
05	Partner	8:54:30	This is true but at least we have the ability to do a little planning lol

**Table:** 

Example C03			
Turn	Interlocutor	Time sent	Message
Turn	Interlocutor	Time sent	Message
01	A	9:28:24	Guess who didnt get funding for next year
02	Partner	9:29:02	[Redacted]!
03	A	9:29:53	Me!
04	Partner	9:30:14	Yayjjj!!
05	Partner	9:30:16	Oh wait
06	A	9:30:26	Beb lol
07	Partner	9:30:32	Is ok babe. How are you feeling about it?

**Table:** 

Example H01			
Turn	Interlocutor	Time sent	Message
01	Friend11	16:39:51	Want to go out at like 2–3?
02	H	16:40:39	Yeah lets do that
03	H	16:41:52	Also again ive never been there for the rafting or hiking or courses so i have no preference or idea what we should do
04	Friend11	17:55:55	Haha ill show you around its all good
05	H	17:57:51	Haha all right so should i just wear workout clothes?
06	Friend11	18:07:43	Yeah that sounds great. About to play our gig so ill text you after. Hope youve had a good day)
07	H	18:11:31	Good luck!!! yeah let me know how it goes!

**Table:** 

Example H02			
Turn	Interlocutor	Time sent	Message
Turn	Interlocutor	Time sent	Message
01	Friend11	12:30:33	How are you?
02	H	16:10:59	Good!! todays gone by so fast
03	Friend11	16:52:34	Yeah todays been a good one, we went to the montreal botanical gardens which sounds boring but it was actually pretty sweet. Getting excited for tonight?
04	H	16:58:48	Haha yea kinda i just have no idea whos coming or when but well see how it goes!!!
05	Friend11	17:06:45	Wish I could be there, hope its alot of fun! what did you and rebecca do today?
06	H	18:06:58	We drove around and then sat at some coffee place and did work then she went to some yoga place and now shes back so were about to start getting things ready
07	Friend11	19:03:31	Gotcha sounds like it was a great day. At dinner ill text you after hope its #lit

**Table:** 

Example H03			
Turn	Interlouctor	Time sent	Turn
01	Friend11	1:21:12	Whats your snap
02	H	1:21:33	Hahahahhh
03	H	1:21:38	[Redacted]
04	Friend11	1:21:46	U Got
05	Friend11	1:21:49	Ok
06	Friend11	1:23:19	But for real, this is random but you are amazing and im really glad we met. Spending time with you is so much fun
07	H	1:27:26	No I know but it was so random how it started and I am too it's gonna suck not being able to hang out for the next week
08	H	1:27:31	I agree
09	Friend11	1:28:25	I meant like me saying that was random haha
10	H	1:28:46	No no I know
11	H	1:29:00	But then I decided to include that little detail
12	Friend11	1:29:01	But yeah im going to miss you. Ill snap or text you when I get to [REDACTED]
13	Friend11	1:29:07	Oh beastttt
14	H	1:29:17	Thank u for that
15	Friend11	1:29:31	Youre very welcome
16	H	1:29:44	And yea snap me

**Table:** 

Example H04			
Turn	Interlouctor	Time sent	Turn
Turn	Interlocutor	Time sent	Message
01	Friend11	19:03:31	How was last night?
02	H	10:49:41	Hahah i mean it was good and fun but i was kinda bored and wanted people to just leave
03	Friend11	10:56:53	Hahaha classicc
04	Friend11	11:04:51	Headed to the train station to go to [REDACTED]

Another explanation may be that H and Friend11’s relational closeness was undergoing a shift. On the day that Friend11 appears to have left town, the two exchanged late-night messages in which Friend11 asked for H’s Snapchat handle, as shown in Example H03. In their pre-study interview, H indicated that they typically message with their most intimate friends on Snapchat, so the addition of Snapchat as another channel for H and Friend11 to communicate through might signal such a potential shift in intimacy. This is further evidence by the message Friend11 sends in turn 06 (“But for real, this is random but you are amazing and im really glad we met. Spending time with you is so much fun”), and H’s response in turn 07. Prior to exchanging closings salutations, the two then agree to send messages through Snapchat and text while Friend11 is out of town, as they do in Examples H02 and H04. In other words, the initial *Haha*s in Examples H02 and H04 may signal the potential change in their friendship. In composing these features more slowly (as well as writing more), it is possible that H was paying greater attention to the text they produced (Biber and Conrad, 2009; Kendall, 2013) in order to engage in more self-monitoring (Hayes, 2012; Bowen and Van Waes, 2020) to be strategic about what they write in sending or responding to messages, and this may have been done in order to maintain the possibility of further advancing their relationship with Friend11. Indeed, when more closely examining the sequence of keystrokes for how H composed the message in turn 02 in Example H04, H initially began composing the message *without* the *Hahah*. H *added* the *Hahah* as part of a message construction repair (Meredith and Stokoe, 2014), so the slower timing process for the *Haha* feature at the initial position (71.8ms) may also reflect greater cognitive effort as H strategically self-monitored their message content—including use of *Haha*—in order to preserve their changing relationship with Friend11.

This closer examination of how participants C and H composed *Haha* or *Lol* at different positions at different speeds offers a window into the complex and variable ways that these individuals may meaningfully manage their relationships through the psychomotor process of inputting text. In particular, this examination suggests that the timing processes of these features may indeed reflect the discursive conditions under which individuals are working, through the socio-technical and even psychomotor mechanisms, to manage facework; and this may correspond with the structural positioning of paralinguistic features that are intended to symbolically manage face. Further, this finding simultaneously demonstrates the value in combining closer qualitative analysis amid the breadth of data obtained from keystroke-logging methods.

## Discussion

This study used keystroke-logging methods in order to examine how paralinguistic variables were composed as part of text-based, mobile communication. Its research questions asked, based on keystroke data, 1) What are the frequencies of occurrence of paralinguistic variables *Haha* and *Lol* in text messaging, and what is their distribution according to turn-taking structures? And, 2) What are the timing processes of *Haha* and *Lol* in text messaging, and how do these timing processes reflect turn-taking structures in asynchronous messaging? This study found that, for the participants that sent either of these features in texting (*N =* 6), pauses before composing *Haha* and *Lol* were significantly greater than for all other kinds of keybursts (see Table 6). Since these features also occurred with consistent, low frequency at initial and terminal turn-taking positions in a message, I suggest a possible interpretation of this finding is that *Haha* and *Lol* are established and discursively salient features for these participants, and the longer pause prior to composing these features suggests these participants are expounding more cognitive resources to carefully attend to how they use these features.

This study also found that, when comparing the speeds at which *Haha* and *Lol* were produced at the starts and ends of messages in SMS and Snapchat (*N =* 132), that these keybursts were produced faster at the start of a message than at the end of the message. A possible explanation for this may be that the different timing processes is reflective of the use of these paralinguistic features to signal different politeness strategies at the start and end of a message. In other words, composing *Haha* or *Lol* at the start of a message may be faster because participants were reacting to the message from their interlocutor's previous turn, whereas composing these features at the end of a message may be slower because participants were self-monitoring their own message to maintain their own positive face.

These findings support theories of cognitive processes of writing, particularly for the processes of producing and monitoring textual production (Hayes, 2012; Leijten et al., 2014), as well as Kendall’s (2013) and Biber and Conrad’s (2009) suggestions that greater attention to producing spoken or written language may affect the timing of those articulatory processes. Further, while this study was concerned with paralinguistic features typically represented in discrete word units, these findings do suggest that, as Eisenstein (2015) posits, that the production of text reflects the residue of spoken language production. As demonstrated by these methods for conducting linguistic analysis from keystroke data, it may therefore prove fruitful to examine morpho-syntactic or morpho-phonological variables to examine the extent to which, as Eisenstein (2015) speculates, this residue permeates to different linguistic levels.

### Limitations

While I maintain that the findings of this study remain promising, I in no way argue these findings are generalizable due to its limitations. Indeed, this was the first study to use LogKey, and one of the first—if not the first—to study keystroke-logging analysis from individuals' personal mobile devices for the period of one week. This study therefore simultaneously functioned as a test of its methodological feasibility, or, to paraphrase the software engineer to helped design LogKey, this study was like a beta test. This study was therefore methodologically limited in two ways: first, in terms the design of LogKey, and in the amount of data yielded from this study. LogKey's design and logging methods could therefore be improved to be more usable, and to log additional records. For example, logging when an application is opened on a device, when SMS messages are transmitted, and when the virtual keyboard appears on a mobile device's screen would all aid in more precisely determining when users might read a received message, and begin to plan composing a response to that message. Further, log records that include information regarding approximate GPS coordinates or gyroscope sensors would provide information regarding whether participants may be in private or public spaces as well as how they might be moving about those spaces.

### Future Directions

In addition to addressing the limitations discussed above, future study could continue to examine the data collected in different ways. For example, analysis of revision patterns could examine whether patterns found by Bowen and Van Waes (2020) may extend to texting, analysis of the timing processes of morpho-phonological features, such as -t/-d or -ing deletion, may examine Einstein’s (2015) observations from Twitter data, and more detailed analysis of *pause bursts* may yield greater insight into how the psychomotor processes of composing on mobile devices may differ from writing on computer-based keyboards (Mangen et al., 2015; Galbraith and Baaijen, 2019). Such analyses would necessarily expand upon the tools used for this study, requiring means to parse keystroke data in order to identify revision bursts and morphological features from keybursts.

At the same time, future study could apply these methods of combining linguistic analysis with keystroke data to examine other mobile writing registers. For example, writing on Twitter, Snapchat, Instagram, or other forms of social media would be valuable not only to examine the writing processes on these registers, but also perhaps also allow examination of emerging text-based linguistic features. Further, as was evident from participants in this study, while text-messaging is predominantly text-based, use of emojis and other visual elements is increasingly valuable, therefore developing means to examine the production of text in conjunction with use of visual symbolic material (see Ilbury, 2018) may paint a different picture than examining text alone. Of course, all of these possible future directions would require not only a more robust version of LogKey, or a similar keystroke logger, but also coordination with other data channels. For example, as Olive and Cislaru (2015) demonstrated, combining corpus-based methods for collected large data sets from Twitter and keystroke-logging methods may offer valuable understanding into how text-based linguistic features are diffused *and* recontextualized by individuals. Such a study would have the ability to more powerfully understand how individuals perform the individual through the social.

### Coda

I hope I have demonstrated the potential value of computational methods such as keystroke logging, especially for examining text-based data. As I have laid out above, the development of keystroke analysis methods for examining text-based data produced through mobile technology offers one possible route to conduct sociolinguistic research in the 21st century. Through such a methodology, linguistic units not just asynchronous artifacts, but may be again seen as evidence of flesh-and-blood processes for articulating language in real-time.

## Data Availability Statement

The datasets generated for this study will not be made publicly available per the privacy, data protection policy, and informed consent process that was approved by the North Carolina State University's Institutional Review Board, data from this study may not be shared.

## Ethics Statement

The studies involving human participants were reviewed and approved by North Carolina State University Institutional Review Board (Protocol 11651). The participants provided their written informed consent to participate in this study.

Written informed consent was obtained from the individual(s) for the publication of any potentially identifiable images or data included in this article.

## Author Contributions

The author confirms being the sole contributor of this work and has approved it for publication.

## Conflict of Interest

The author declares that the research was conducted in the absence of any commercial or financial relationships that could be construed as a potential conflict of interest.

## Footnotes

1 I use the term *digital* to simultaneously reference the fact that writing is often composed through both computational technologies as well as inputted via digits on human hands.

2 Developed by Leijten & Van Waes (2013).

3 LogKey was designed and implemented by Nicholas Miano over several months while in consultation with the author. Dr. William Enck, Associate Professor of Computer Science at North Carolina State University, conducted code review to ensure that the app met stringent security standards in addition to a review by university IRB.

4 The Informed Consent Script informed participants’ interlocutor that the participant was involved in a study that would be collecting their keystroke data and downloading SMS conversations from their phones, if the interlocutor consented in writing as well. The script further explained that if the interlocutor consented, messages sent from them would be used to contextualize content that the participant composed.

5 Hadley Wickham, Romain François, Lionel Henry and Kirill Müller (2018). dplyr: A Grammar of Data Manipulation. R package version 0.7.5. https://CRAN.R-project.org/package=dplyr
